# Ozone Treatment Inhibited the Blue Mold Development and Maintained the Main Active Ingredient Content in *Radix astragali* Infected by *Penicillium polonicum* Through Activating Reactive Oxygen Species Metabolism

**DOI:** 10.3390/jof11060402

**Published:** 2025-05-23

**Authors:** Jihui Xi, Qili Liu, Qingru Zhang, Zhiguang Liu, Huali Xue, Yuqin Feng

**Affiliations:** 1 College of Science, Gansu Agricultural University, Lanzhou 730070, China; xijihui2022@163.com (J.X.); liuzhiguang04@sina.com (Z.L.); 2 College of Food Science and Engineering, Gansu Agricultural University, Lanzhou 730070, China; lql744607@163.com; 3 Science and Technology Development Institute, Gansu Agricultural University, Lanzhou 730070, China; qingru31@163.com; 4 Agricultural Product Storage and Processing Research Institute, Gansu Academy of Agricultural Sciences, Lanzhou 730070, China

**Keywords:** *Radix astragali*, *Penicillium polonicum*, patulin, ozone, active ingredient, postharvest diseases

## Abstract

*Radix astragali* is a homologous plant of medicine and food with a variety of health benefits. However, our previous study showed that blue mold, caused by *Penicillium polonicum*, is the most important postharvest disease of fresh *R. astragali* during storage. Ozone, as a strong oxidizing agent, can effectively control the occurrence of postharvest diseases in fruits and vegetables. Nevertheless, there are few research studies on the effect of ozone-treated fresh Chinese traditional medicine. In this study, we investigated the effect of ozone gas on the postharvest blue mold development, mycotoxin accumulation, and main active component contents in fresh *R. astragali* infected by *P. polonicum*, and analyzed the possible action mechanism on ROS metabolism. The result indicates that ozone application significantly inhibited the development of postharvest blue mold caused by *P. polonicum* infection, reduced the disease incidence, disease index, and weight loss rate, maintained the main active ingredients in fresh *R. astragali* by activating ROS metabolism, enhanced the antioxidant enzymatic activity, thus avoiding oxidative damage caused by excessive ROS accumulation, and maintained the integrity of the cell membrane, ultimately controlling the occurrence of blue mold of *R. astragali*. Moreover, ozone treatment also maintained the contents of the main active ingredients in *R. astragali* before 14 d during *P. polonicum* infection. In addition, the amount of active ingredients of astragaloside I, calycosin-7-glucoside, and ononin in the ozone-treated group was higher than that in the control group during the storage period. We speculate that, under the action of ozone, astragaloside IV was converted into astragaloside II by oxidative modification and astragaloside II was further oxidized to astragaloside I, resulting in the accumulation of astragaloside I. Similarity, the hydrogen atoms (-H) on the benzene ring in formononetin were oxidized to phenolic hydroxyl groups (-OH) to generate calycosin, which was further converted into calycosin-7-glucoside, resulting in calycosin-7-glucoside accumulation. This study will provide the theoretical basis for ozone commercial application to control the occurrence of postharvest diseases of *R. astragali*.

## 1. Introduction

*Radix astragali*, called “Huang-qi” in China, is a homologous plant of medicine and food with a variety of health benefits that has been utilized for over two millennia in China [1]. The pharmacopoeia of the People’s Republic of China [2] suggests that *R. astragali* is regarded as a plant of *Astragalus membranaceus* in the leguminous family whose main active ingredients are polysaccharides, amino acids, astragaloside I, calycosin-7-glucoside, ononin, and trace elements and other components according to modern medical research [3], with hepatoprotective, expectorant, and diuretic effects, and an antitumor [4], anti-aging [5], anti-inflammatory [6], antiviral [7], antioxidant [8] and immune-system-promoting function [9]. Zhang Li et al. [10] determined 17 active components in *R. astragali* from different origins based on UPLC-TQ-MS, including isoastragaloside IV, astragaloside IV, astragaloside III, astragaloside II, soyasaponin I, isoastragaloside II, astragaloside I, isoastragaloside I, calycosin-7-glucoside, genistein, ononin, methylnissolin-3-O-glucoside, cyclosin, genistein, formononetin, methylnissolin, and isomucronulatol. Astragaloside, also known as astragaloside IV, is one of the important active components of *R. astragali*. Astragaloside regulates signaling pathways in vivo and has a variety of pharmacological effects, including anti-inflammatory, antifibrotic, antioxidative stress, antidiabetic, and antitumor effects [11]. Calycosin-7-O-β-D-glucoside is a monomer component with a wide range of pharmaceutical activities, belonging to the isoflavonoids class of compounds, with multiple medicinal values, such as antioxidant, antimicrobial, antiviral, antitumor, melanin-formation-inhibiting, and immunomodulatory activities [12].

With the increase in the planting area of *R. astragali*, the pre- or postharvest diseases caused by fungal infection have become increasingly apparent—for instance, root rot caused by *Fusarium solani* [13], gray mold rot caused by *Botrytis cinerea* [14], and blue mold caused by *Penicillium oxalicum* [15]—which all seriously impact the yield and quality of *R. astragali*. However, previous studies on *R. astragali* diseases have mainly focused on the field growth period (pre-harvest) or dried decoction pieces (postharvest), and there are few reports on postharvest disease, mycotoxin accumulation, and especially the change in the main active ingredients during the postharvest storage of fresh *R. astragali*.

*R. astragali* is a Gansu-specific medicinal herb that is harvested in late fall, low temperature, and high humidity. The moisture content of fresh *R. astragali* is relatively high and its root tissue is rich in polysaccharides, proteins, and so forth, which favor pathogenic fungi growth. At the same time, the storing technology for fresh traditional herbal medicine in Gansu Province is quite underdeveloped, contributing to postharvest decay, which ultimately seriously impacts the quality of *R. astragali* due to fungal infection and mycotoxin contamination. It was reported that the losses due to pathogenic fungi contamination are approximately 15–25% annually [16]. The inadequate control of temperature and humidity in the postharvest storage of *R. astragali* can lead to an infestation of fungi, leading to disease, where the fungi are able to metabolize and produce mycotoxins, resulting in serious losses in the yield and economy of *R. astragali*, as well as serious health hazards to consumers [14,15]. Preliminary research by our group found that *Penicillium polonicum*, *Trichothecium roseum*, and *Fusarium equiseti* are the main pathogen species causing postharvest diseases in *R. astragali* [17], with *Penicillium polonicum*, which can be metabolized in the host to produce carcinogenic, teratogenic, and mutagenic patulin (PAT), being the most serious [18]. At present, fresh *R. astragali* is mainly subjected to sulfur fumigation after harvest; however, excessive SO_2_ not only leads to sulfur dioxide residue but also causes the destruction of some active ingredients, thereby directly reducing or changing its medical efficacy [19]. Therefore, an effective, green, and eco-friendly strategy is urgently proposed.

Ozone (O_3_), as a strong antioxidant, is confirmed as a fresh-keeping agent by the Food and Drug Administration due to its outstanding antimicrobial effect [20], and is extensively applied to manage postharvest diseases for agricultural products, especially for fruit and vegetables [21]. Moreover, due to its strong oxidizing properties, O_3_ is effective at inhibiting fungal growth and degrading mycotoxins [22]. The management of ozone for postharvest plant disease is attributed to two aspects: on the one hand, ozone application directly suppresses fungal growth and reduces plant decay; for instance, Matłok et al. [23] indicated that O_3_ application managed microorganism development on the saskatoon berries’ surface and effectually reduced disease during storage; on the other hand, ozone activates the reactive oxygen species (ROS) production system and stimulates ROS metabolism. Luo et al. [24] found that ozone treatment could control the occurrence of postharvest diseases in kiwifruit by increasing antioxidant enzyme activities in the host. However, research on postharvest disease and mycotoxin accumulation, especially the contents of main active components in Chinese traditional herbs resulting from ozone treatment, has not been well documented.

In this study, we analyzed the effects of ozone treatment on the postharvest blue mold development, mycotoxin accumulation, and main active component contents in fresh *R. astragali* infected by *Penicillium polonicum*, and analyzed the possible action mechanism on ROS metabolism.

## 2. Materials and Methods

### 2.1. Preparation of Spore Suspension

*Penicillium polonicum* was previously isolated and purified from *R. astragali* with typical symptoms of blue mold and was identified by our research group [17]. *P. polonicum* was cultured on potato dextrose agar (PDA) for 7–10 d prior to further use. A spore suspension was then obtained and a concentration of 1 × 10^6^ spores mL^−1^ was modified using the method described by Li et al. [25].

### 2.2. Preparation of Ozone Gas and Treatment of Radix astragali (RA)

An ozone generator (OSAN CFG, Aoshan Environmental Protection Technology Co., Ltd., Dalian, China) was used to produce gaseous ozone, and the ozone concentration was adjusted to 2.0 mg L ^−1^ according to Li et al. [25].

Fresh *R. astragali* was collected from Min County, Gansu Province. After careful selection, fresh *R. astragali* roots of similar size with no obvious mechanical damage or pests were transferred to the Chemical Biology Laboratory, then disinfected by immersion in 1% NaClO for 15 min, and then washed three times with distilled water. The prepared spore suspension was evenly inoculated onto the surface of *R. astragali* (at a concentration of 1 × 10^6^ spores mL^−1^ and a volume of 2 mL), and the inoculated *R. astragali* was placed in a sterile bottle of 2 L for ozone treatment, where the treatment time was 0, 1, and 2 h. It was then kept in sterile preservation bags and then placed at room temperature for 7, 14, 28, 42, and 56 d (23~27 °C and 40% RH). The collected samples of *R. astragali* were immediately frozen in liquid nitrogen and kept at −80 °C.

### 2.3. Effect of Ozone Treatment on Weight Loss Rate of Fresh R. astragali

The effect of ozone treatment on the weight loss of the fresh *R. astragali* was based on the method of Liu et al. [26], and the weight loss rate was calculated according to the following Formula (1).

(1) $$W=\frac{W_{1}-W_{2}}{W_{1}}\times 100\%$$

In the formula, W is the weight loss rate, %; W_1_ is the gram of *R. astragali* before storage, g; W_2_ is the gram of *R. astragali* after storage, g.

### 2.4. Effect of Ozone Treatment on Disease Incidence of Fresh R. astragali

The ozone treatment method was based on the above Section 2.2 [25]. The disease incidence and disease index were counted and calculated by referring to our previous publication [20].

### 2.5. Effect of Ozone Application on the Patulin Production in Fresh R. astragali

The ozone treatment method was based on the above Section 2.2, and the content of patulin (PAT) in *R. astragali* was determined based on our previous publication by Lv [27].

### 2.6. Effect of Ozone Treatment on the Main Active Ingredients of Fresh R. astragali

The ozone treatment method was based on the above Section 2.2. The ten kinds of main active components in the fresh *R. astragali* are astragaloside I, astragaloside II, ononin, astragaloside III, astragaloside IV, calycosin, 7,2′-dihydroxy-3′,4′-dimethoxyisoflavan, calycosin-7-glucoside, formononetin, and 3-hydroxy-9,10-dimethoxypterocarpan. Regarding the chromatographic separation conditions, the chromatographic column was a Phenomenex Kinetex C18 column (100 mm × 2.1 mm, 1.7 μm) and the mobile phases contained 0.1% formic acid (A) and acetonitrile (0.1% formic acid, *v*/*v*) (B). The separation gradient was as follows: 0–3 min, 90% A, 3–10 min, 90–65% A,10–20 min, 65–0% A, 20–22.5 min, 0–90% A, 22.5–25 min, 90% A. The flow rate was 0.3 m/min and the injection volume was 2 μL. The mass spectrometry conditions were as follows: ionization mode: ESI and negative; ion spray voltage: 4.5 kV; ionization temperature: 500 °C; declustering potential (DP): −100 V; collision energy (CE): −10 V; MSI range: 100–1200 (*m*/*z*).

### 2.7. Malonaldehyde (MDA) Content Assay

The determination of MDA was according to the method of Yang et al. [28].

### 2.8. The Production Rate of O_2_^−^· and H_2_O_2_ Content Assay

The H_2_O_2_ content and production rate of O_2_^−^· were determined by referring to Bao et al. [29]. The H_2_O_2_ content was indicated as mmol g^−1^ FW. The O_2_^−^· production rate was indicated as µmol min^−1^ g^−1^ FW (fresh weight).

### 2.9. Enzymatic Activities Assay

#### 2.9.1. Assay of the Enzymatic Activities Involved in ROS Production

The activities of NADPH oxidase (NOX) and superoxide dismutase (SOD) were assayed by adopting the kits Beijing Solarbio Science & Technology Co., Ltd. (Beijing, China), BC0630 and BC5165, and the units of the NOX and SOD activities were expressed as U g^−1^ FW.

#### 2.9.2. Assay of the Enzymatic Activities Involved in ROS Depletion

The activities of peroxidase (POD) and catalase (CAT) were assayed by adopting the kits Beijing Solarbio Science & Technology Co., Ltd., BC0090 and BC0200, and the units of POD and CAT activities were expressed as U g^−1^ FW.

#### 2.9.3. Assay of the Key Enzymatic Activities Involved in AsA-GSH Cycle

The activities of ascorbic peroxidase (APX) and dehydroascorbate reductase (DHAR) were assayed by adopting the kits Beijing Solarbio Science & Technology Co., Ltd., BC0220 and BC0660, and the units of APX and GR activities were expressed as U g^−1^ FW. The activities of monodehydroascorbate reductase (MDHAR) and glutathione reductase (GR) were assayed according to the kits Beijing Solarbio Science & Technology Co., Ltd., BC0650 and BC1160. The activities of MDHAR and GR were indicated as U g^−1^ FW.

### 2.10. Statistical Analysis

All experiments were implemented in triplicate. Data were expressed as means ± standard errors. The figures were created with Origin 2021 (Northampton, MA, USA). SPSS 22.0 was used to process the difference significance (* *p* < 0.05, ** *p* < 0.01, *** *p* < 0.001).

## 3. Results

### 3.1. Ozone Treatment Delayed Water Loss in Fresh R. astragali

**Figure 1:**
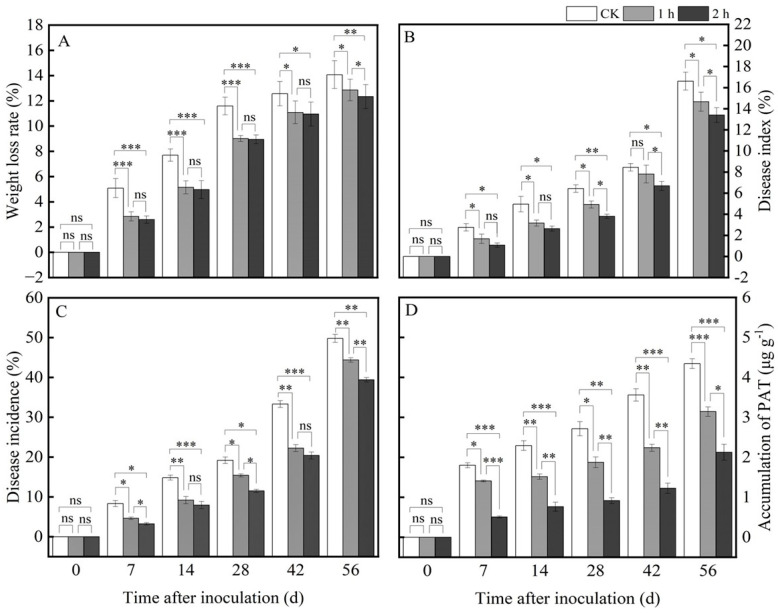
Effect of ozone treatment on weight loss rate (**A**), disease index (**B**), disease incidence (**C**) and PAT accumulation (**D**) of fresh *R. astragali*. Values in the figures were shown as the means ± standard error (n = 3). The asterisk means a significant difference (* *p* < 0.05, ** *p* < 0.01, *** *p* < 0.001, ns stands for not significant) between sample groups at the same storage day.

As we know, water loss for fresh agricultural products is one of the most common phenomena due to transpiration and respiration. The weight loss rate in fresh *R. astragali* increased during the whole storage in both treated and control groups (Figure 1A). Especially at 0–28 d, the weight loss rate of the fresh *R. astragali* increased rapidly; however, from the 28th day to 56th day, it increased slowly. In general, ozone application delayed the increase in the weight loss rate of RA. For instance, on the 28th day, the weight loss rates in the 1 h and 2 h ozone-treated groups were 9.01% and 8.94%, respectively, while it was 11.59% in the control group.

### 3.2. Ozone Treatment Inhibited the Blue Mold Development and Patulin Accumulation in Fresh R. astragali

Ozone application significantly inhibited the development of blue mold in fresh *R. astragali*, and the longer the ozone-treated time, the more obvious the effect observed.

On the 56th day of storage, after ozone treatment for 1 h and 2 h, the disease incidence was 44.36 ± 0.55% and 39.42 ± 0.55%, and the disease index was 14.66 ± 0.90% and 13.4 ± 0.70%, respectively, while the disease index and disease incidence in the control group were 16.61 ± 0.85% and 49.8 ± 1%, respectively (Figure 1B,C). Similarly, ozone treatment remarkedly reduced PAT accumulation in fresh *R. astragali*. For instance, on the 56th day after treatment, the PAT content for ozone treatment for 2 h was reduced by 51.15% compared with the control (Figure 1D).

### 3.3. Ozone Treatment Maintained the Main Active Ingredient Contents of Fresh R. astragali

**Figure 2:**
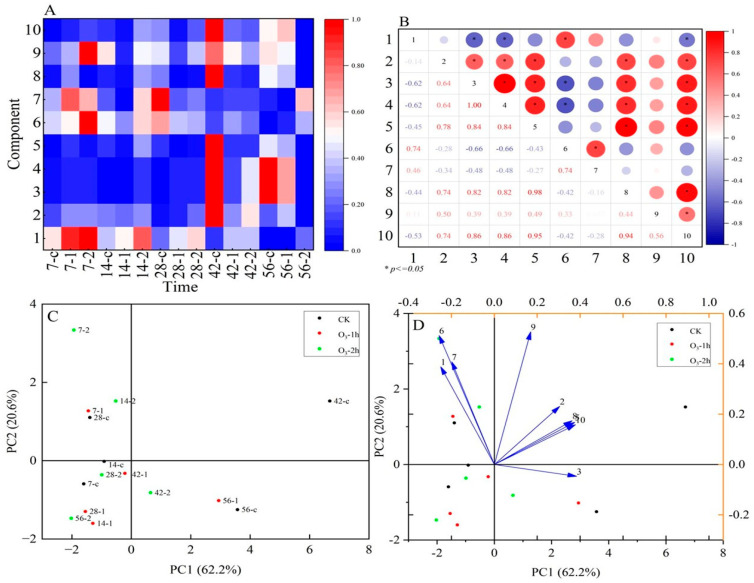
Heatmap visualization of the main active ingredient in the O_3_-treated and control groups infected by *P. polonicum* in fresh *R. astragali* (**A**); correlation analysis of the main active ingredient in the O_3_-treated and control groups infected by *P. polonicum* in fresh *R. astragali* (**B**); scoring plot (**C**) and loading plot (**D**) of O_3_ treatment on principal component analysis of pharmacodynamic components of fresh *R. astragali*.

The ten main active ingredients of astragaloside I, astragaloside II, ononin, astragaloside III, astragaloside IV, 7,2′-dihydroxy-3′,4′-dimethoxyisoflavan, calycosin, calycosin-7-glucoside, formononetin, and 3-hydroxy-9,10-dimethoxypterocarpan were determined by HPLC-MS in the fresh *R. astragali*. The contents of the ten main active ingredients in the control and ozone-treated groups are presented as supporting information Table S1. In general, regardless of the treatment or control, the contents of astragaloside I, calycosin-7-glucoside, and ononin decreased gradually with the prolongation of storage time, while the contents of astragaloside II, astragaloside III, astragaloside IV, formononetin, calycosin, 7,2’-dihydroxy-3’,4’-dimethoxyisoflavan, and 3-hydroxy-9,10-dimethoxypterocarpan increased with the prolongation of storage time. Moreover, ozone application significantly maintained the contents of the 10 components before 14 d compared with the control group. However, from the 42nd to 56th day, the contents of astragaloside I, calycosin-7-glucoside, and ononin in the ozone-treated group were higher than those in the control group, and the contents of the other components were lower than in the control group (supporting information Table S1). Correlation analysis of the 10 active ingredients of *R. astragali* indicated that astragaloside I exhibited a positive correlation with calycosin-7-glucoside. 7,2′-dihydroxy-3′,4′-dimethoxyisoflavan and 3-hydroxy-9,10-dimethoxypterocarpan exhibited a positive correlation with astragaloside II, astragaloside III, astragaloside IV, and calycosin. Calycosin-7-glucoside was positively correlated with ononin, as well as with astragaloside II, astragaloside III, and astragaloside IV (Figure 2A,B). The score plot (Figure 2C) and factor load plot (Figure 2D) of *R. astragali* suggest that, with a prolonged storage time, the larger the characteristic value, the higher the content of *R. astragali* determined, among which, the control group on the 42nd day has the highest content of principal components. At 0–28 d, the principal component in the control group had a high similarity with the ozone treatment group, and a significant positive correlation was observed in the content change. In addition, among them, the positive correlation between the 1 h and 2 h ozone-treated groups on the 7th day and 1 h ozone-treated group on the 14th day was the strongest. On the 56th day, ozone treatment also had a high similarity with the control group, and its content change presented a positive correlation.

### 3.4. Ozone Treatment Activated ROS Metabolism and Kept Redox Homeostasis in Fresh R. astragali

#### 3.4.1. Ozone Treatment Decreased MDA Content in Fresh *R. astragali*

**Figure 3:**
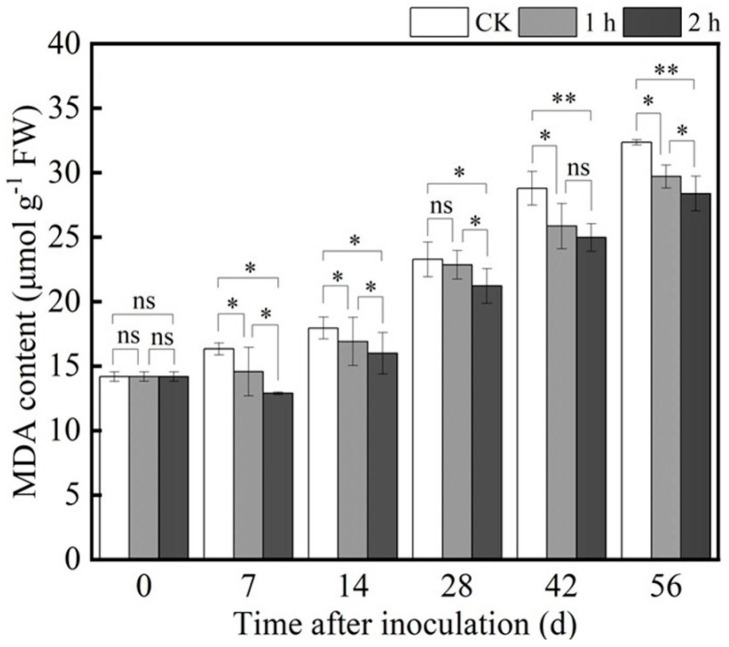
Effects of ozone treatment on MDA content of fresh *R. astragali*. Values in the figures are shown as the means ± standard error (n = 3). The asterisk means a significant difference (* *p* < 0.05, ** *p* < 0.01, ns stands for not significant) between sample groups at the same storage day.

MDA content is commonly used to evaluate the degree of lipid peroxidation for cell membranes and the integrity of the cell membrane. *P. polonicum* infection led to an increase in MDA content in fresh *R. astragali*, and the MDA content was generally enhanced with prolonged storage time. Nevertheless, ozone treatment significantly reduced MDA content; for instance, on the 56th day of storage, compared with the control, the MDA content in ozone-treated *R. astragali* decreased by 8.15% and 12.27% after treatment for 1 h and 2 h, respectively (Figure 3). In fact, the lower MDA content is attributed to the ROS metabolism activation and redox homeostasis maintenance.

#### 3.4.2. Ozone Treatment Decreased the O_2_^−^· Production Rate and H_2_O_2_ Content in Fresh *R. astragali*

**Figure 4:**
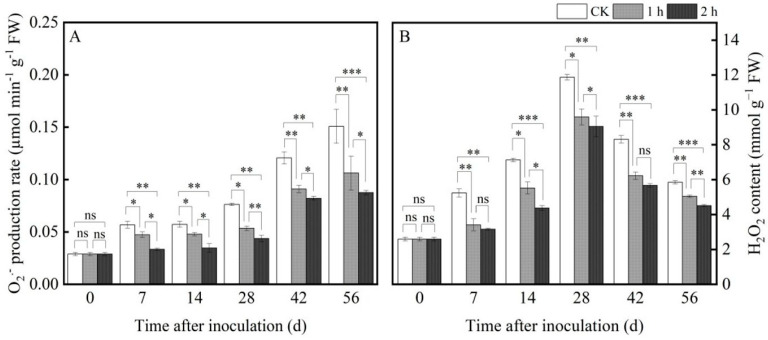
Effects of ozone treatment on O_2_^−^·production rate (**A**) and H_2_O_2_ content (**B**) in fresh *R. astragali*. Values in the figures are shown as the means ± standard error (n = 3). The asterisk means a significant difference (* *p* < 0.05, ** *p* < 0.01, *** *p* < 0.001, ns stands for not significant) between sample groups at the same storage day.

Ozone application remarkedly reduced the O_2_^−^· production rate and H_2_O_2_ content during the whole storage process. The O_2_^−^· production rate increased with the extension of storage time and reached its peak on the 56th day of storage. On the 56th day, compared with the control group, the O_2_^−^· production rate decreased by 26% and 42% after ozone treatment for 1 h and 2 h, respectively (Figure 4A). However, the content of H_2_O_2_ increased first and then decreased during the storage time, reaching its peak on the 28th day. Compared with the control group, the H_2_O_2_ content was reduced by 13.7% and 22.77% after 1 h and 2 h of ozone fumigation, respectively (Figure 4B).

#### 3.4.3. Ozone Treatment Increased the Activities of ROS Metabolism-Related Enzymes

**Figure 5:**
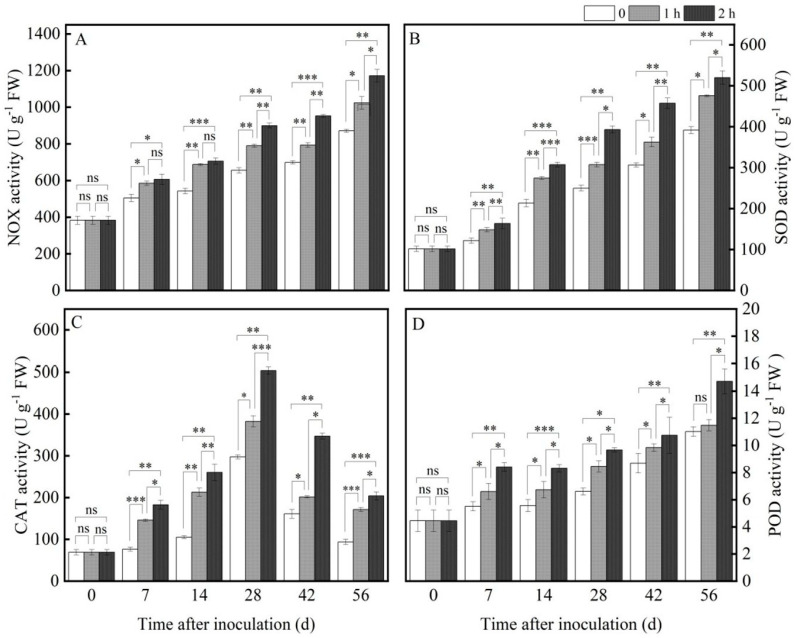
Effects of ozone treatment on the activities of NOX (**A**), SOD (**B**), CAT (**C**), and POD (**D**) in fresh *R. astragali*. Values in the figures are shown as the means ± standard error (n = 3). The asterisk means a significant difference (* *p* < 0.05, ** *p* < 0.01, *** *p* < 0.001, ns stands for not significant) between sample groups at the same storage day.

ROS is one kind of stress response mechanism in host plants when exposed to unfavorable environmental conditions. However, an excessive accumulation of ROS can result in oxidative stress, leading to cellular damage and even death. NOX (NADPH oxidase) is the main regulator enzyme of ROS production, and acts as an electron donor to convert extra-membrane O_2_ to O_2_^−^·. NOX activity continued to increase with prolonged storage time after inoculation, and ozone treatment significantly increased NOX activity; for instance, after ozone treatment for 2 h, the NOX activity was 34.47% higher than that for the control on the 56th day (Figure 5A). In order to cope with oxidative stress due to excessive ROS accumulation and maintain the redox homeostasis, plants have developed a corresponding and adaptive set of adjusting mechanisms to balance the ROS in organisms. The function of SOD is to catalyze O_2_^−^· disproportionation to generate H_2_O_2_ and O_2_. Then, POD and CAT can convert H_2_O_2_ into H_2_O and O_2_. As shown in Figure 5B–D, both SOD and POD activities gradually improved with the prolonged ozone treatment time, CAT activity increased first and then reduced with prolonged preservation time, and ozone treatment significantly increased their activities. The activities of SOD and POD were enhanced by 24.69% and 25.13% on the 56th day after ozone treatment for 2 h compared with the control. CAT activity increased by 41% on the 28th day after ozone treatment for 2 h compared with the control.

#### 3.4.4. Ozone Treatment Increased the Activities of the Enzymes Involved in AsA-GSH Cycle

**Figure 6:**
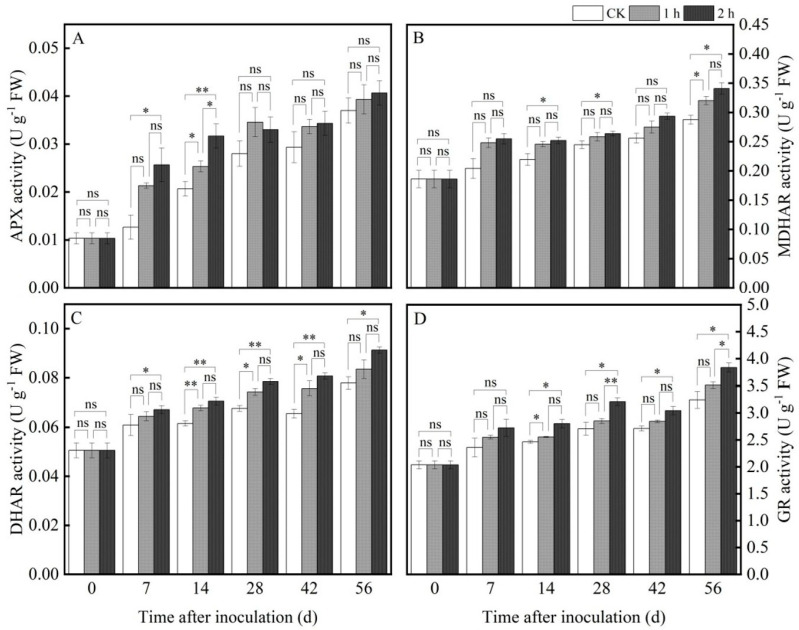
Effects of ozone treatment on the activities of APX (**A**), MDHAR (**B**), DHAR (**C**), and GR (**D**) in fresh *R. astragali*. Values in the figures are shown as the means ± standard error (n = 3). The asterisk means a significant difference (* *p* < 0.05, ** *p* < 0.01, ns stands for not significant) between sample groups at the same storage day.

In addition to ROS metabolism, the AsA-GSH cycle also plays a crucial role in scavenging excessive ROS. The key enzymes (such as APX, MDHAR, GR, and DHAR) that are involved in the AsA-GSH cycle play crucial roles in maintaining the ascorbic acid (AsA) and glutathione (GSH) content. The four enzymes are also the regulator of redox balance in plants. In our study, the activities of APX, MDHAR, DHAR, and GR increased gradually with the prolongation of preservation time in both ozone-treated and control groups. Ozone treatment significantly increased these enzymatic activities; for instance, the activities of APX, MDHAR, DHAR, and GR improved by 9.8%, 15.54%, 11.11%, and 15.43% after 2 h ozone treatment, respectively, on the 56th day compared with the control group (Figure 6A–D). The above results suggest that ozone treatment increased the activity of enzymes related to the ROS metabolic pathway, thereby activating ROS metabolism and maintaining redox homeostasis in fresh *R. astragali*.

## 4. Discussion

In the present study, the results suggest that ozone treatment significantly reduced the weight loss rate and disease incidence, inhibited the development of the blue mold and accumulation of PAT, and maintained the main active ingredients in postharvest fresh *R. astragali*. The longer the ozone treatment time, the more noticeable the effect observed. This result is consistent with the report of Ong et al. [30], who pointed out that ozone treatment inhibited papaya anthracnose caused by *Colletotrichum gloeosporioides*. Li et al. [25] also suggested that ozone application not only suppressed the fusarium rot development of potato tubers but also reduced diacetoxyscirpenol accumulation.

The ozone effects controlling for weight loss rate, disease development, and mycotoxin accumulation were mainly attributed to the maintenance of the cell membrane integrity of the host. As we know, MDA is one of the products of membrane peroxidation decomposition whose content is often adopted to reflect the degree of membrane peroxidation and damage. In our study, ozone treatment significantly inhibited the increase in MDA content in fresh *R. astragali*, better maintained the integrity of the cell membrane (Figure 3), alleviated the infection of *P. polonicum* in the fresh *R. astragali* tissue, reduced the incidence rate and disease index, and inhibited the development of blue mold of *R. astragali*. Moreover, ozone treatment alleviated the weight loss of the fresh *R. astragali* to a certain extent, which is attributed to ozone treatment reducing the cell membrane permeability by decreasing MDA content and inhibiting the respiratory intensity, ultimately resulting in stomatal closure of surface tissue of the fresh *R. astragali* [31].

The maintenance of the cell membrane integrity and decreased MDA content were mainly attributed to the activation of the antioxidant defense system, which is responsible for removing excess ROS. In this study, ozone treatment effectively activated the antioxidant defense system and improved the antioxidant enzymatic activities of the host, thereby improving the disease resistance of *R. astragali*. A similar result was reported by Piechowiak et al. [32] in blueberry. In this study, when the pathogen invaded the tissue of *R. astragali*, a large amount of ROS was detected at the site of tissue infection, resulting in increasing the production rate of O_2_^−^· and H_2_O_2_ content, destroying the balance of ROS in the tissue, and causing lipid peroxidation of the cell membrane of RA, leading to oxidative damage and accelerating the invasion of the pathogen, thus accelerating the decay and deterioration of *R. astragali*. Ozone treatment significantly reduced the production rate of O_2_^−^· and H_2_O_2_ content. Lu et al. [33] also indicated that ozone treatment reduced the production rate of O_2_^−^· and H_2_O_2_ content in apricot fruit, as well as effectively managing their disease development. The balance of ROS production and the scavenging system is an important defense means for plants to maintain normal growth and resist external stress during system evolution [34]. There is an active oxygen scavenging system in plants that can scavenge the excessive ROS produced by external stress and maintain normal metabolism of the plants. NOX, SOD, CAT, and POD are the main ROS antioxidant enzymes. NOX is the main producer of O_2_^−^·, and SOD catalyzes O_2_^−^ disproportionation to produce H_2_O_2_ and O_2_, which are the first to resist oxidative stress. CAT and POD can further decompose H_2_O_2_ into H_2_O and O_2_, thereby avoiding ROS damage. CAT, a marker enzyme of peroxisome, mainly exists in the peroxisome of cells and catalyzes the decomposition of H_2_O_2_ into H_2_O and O_2_. At the same time, POD can catalyze many reactions and has the function of eliminating H_2_O_2_ [35]. In this experiment, the activities of NOX, SOD, CAT, and POD improved with the extension of preservation time, and the activities after ozone treatment were higher than in the control (Figure 5A–D). In fact, ozone treatment could up-regulate the activity of NOX; we speculate that ozone may target a binding membrane protein on the cell membrane, which will lead to the internal flow of extracellular Ca^2+^ and an increase in intracellular Ca^2+^ concentration. Calcium-dependent protein kinase (CDPK) is activated by recognizing and binding intracellular Ca^2+^, and correspondingly activates NOX activity by phosphorylation, which ultimately leads to the generation of O_2_^−^·. Ozone application activated the ROS scavenging-related enzyme activities in the tissues of *R. astragali*, effectively reduced the production rate of O_2_^−^· and H_2_O_2_ content in the tissues of *R. astragali* (Figure 4A,B), balanced the antioxidant system in the tissue of *R. astragali*, reduced the content of MDA, and decelerated the degree of membrane lipid peroxidation. This result is consistent with that of Li et al. [36].

In addition, the AsA-GSH cycle is also an important regulatory system for scavenging ROS in plants. APX, as an important enzyme for ROS scavenging, plays an important role in maintaining ROS balance in vivo. Under the action of ascorbate, APX reduces H_2_O_2_ to H_2_O through an ASA-GSH cycle reaction, thus alleviating the harm to the plant due to a high concentration of ROS. Furthermore, APX, as the first and a crucial enzyme in the AsA-GSH cycle, specifically catalyzes the reaction of AsA with H_2_O_2_ to produce monodehydroascorbate (MDHA). Dehydroascorbate reductase (DHAR) employs the electrons generated from GSH to reduce dehydroascorbic acid (DHA) and provide it to AsA. GSH is oxidized by DHAR to produce GSSG. With the help of GR, oxidized glutathione (GSSG) is subsequently changed to GSH, increasing the antioxidant level of plant. The results of the current research indicate that the activities of APX, MDHAR, DHAR, and GR increased after ozone treatment and were markedly higher than those in the control (Figure 6A–D), suggesting that APX and GR were involved in the removal of H_2_O_2_ in *R. astragali*. A similar result was reported by Lu et al. [37], who confirmed that ozone treatment increased the activity of ASA-GSH-cycle-related enzymes in cantaloupe fruit.

**Figure 7:**
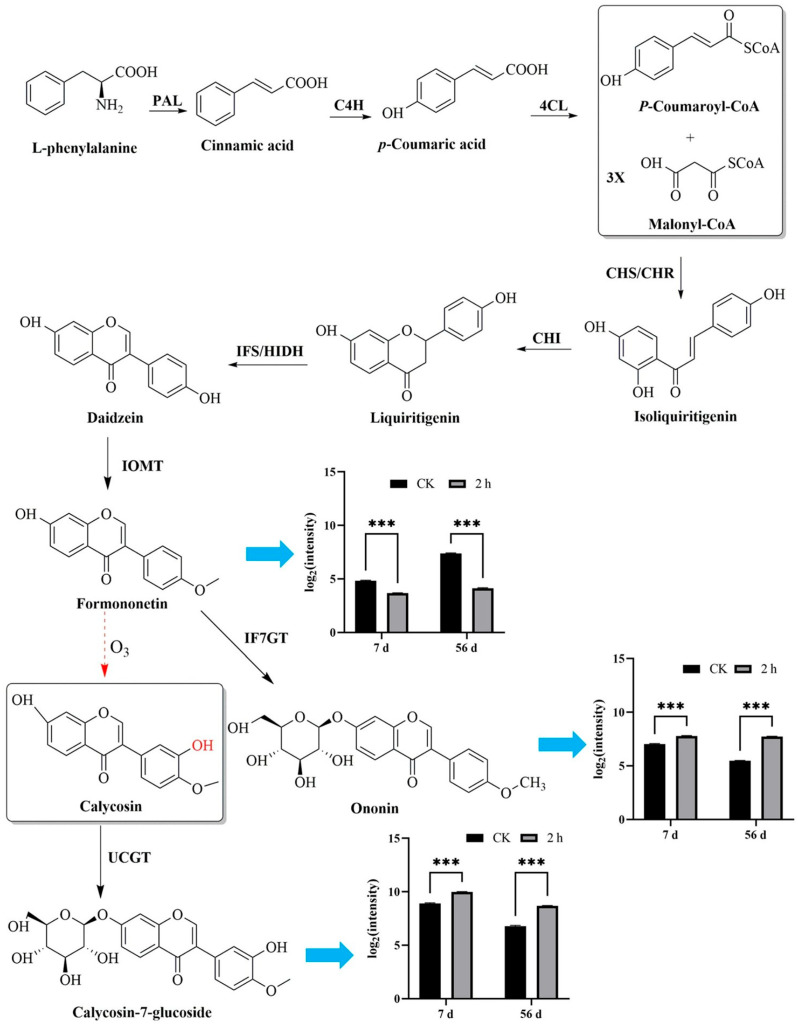
Biosynthesis pathway and mutual transformation of *R. astragali* flavonoids. Values in the figures are shown as the means ± standard error (n = 3). The asterisk means a significant difference (*** *p* < 0.001) between sample groups at the same storage day.

The ten active components of astragaloside I, astragaloside II, ononin, astragaloside III, astragaloside IV, 7,2′-dihydroxy-3′,4′-dimethoxyisoflavan, calycosin, calycosin-7-glucoside, formononetin, and 3-hydroxy-9,10-dimethoxypterocarpan were found in the fresh *R. astragali* by UPLC-MS (Supplementary information Table S1). With the prolongation of storage time, the contents of astragaloside II, astragaloside III, astragaloside IV, formononetin, calycosin, 7,2′-dihydroxy-3′,4′-dimethoxyisoflavan, and 3-hydroxy-9,10-dimethoxypterocarpan showed an upward trend, and the ozone treatment group had higher contents than the control group in the first 14 d (Supplementary information Table S1). It was interesting that the three active ingredients of astragaloside I, calycosin-7-glucoside, and ononin gradually decreased in both the ozone-treated and control groups. With the extension of storage time, the three active ingredients especially sharply declined in the control; however, ozone treatment remarkably decelerated the reduction in the three active ingredients. The reason for this is that the main active ingredients of calycosin, calycosin-7-glucoside, ononin, and formononetin in fresh *R. astragali* are flavonoid compounds. Coumaroyl-CoA is synthesized by the phenylpropanoid pathway, which is an important precursor for the biosynthesis of flavonoids [38]; then, formononetin is synthesized through the flavonoid biosynthesis pathway, which can be oxidized to ononin and transformed into calycosin. Calycosin is further glycosylated to calycosin-7-glucoside. In this study, the content of calycosin-7-glucoside increased significantly and the content of formononetin decreased after ozone treatment. This may be due to the fact that, under the action of ozone, the hydrogen atoms (-H) on the benzene ring in formononetin are oxidized to phenolic hydroxyl groups (-OH) to generate calycosin, which is further converted into calycosin-7-glucoside, resulting in calycosin-7-glucoside accumulation. Moreover, ozone treatment also led to an increase in the content of ononin (Figure 7).

**Figure 8:**
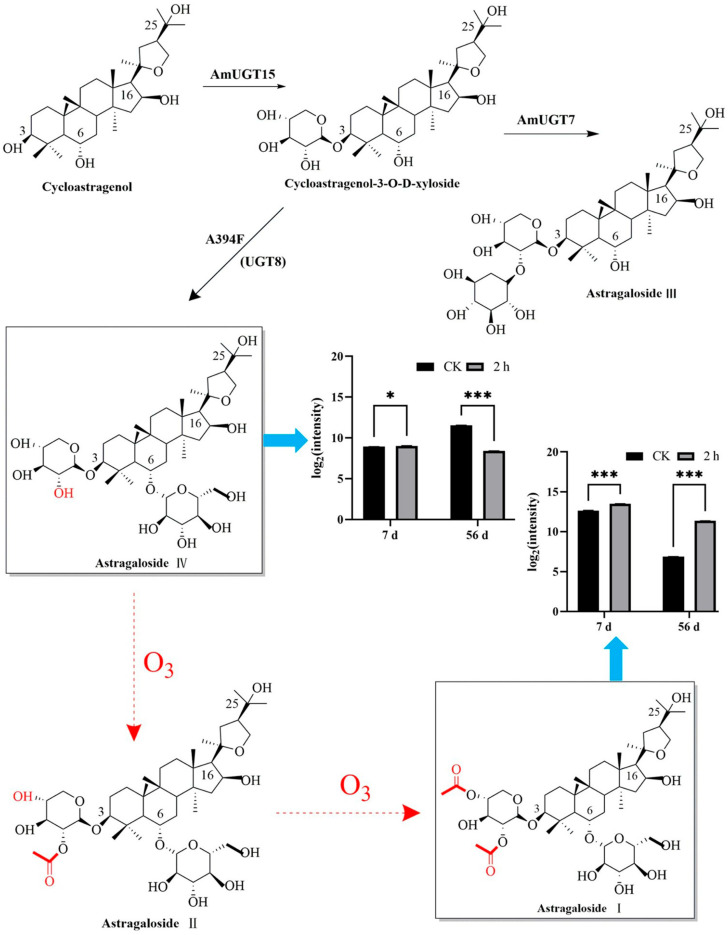
Biosynthesis pathway and mutual transformation of astragaloside. Values in the figures are shown as the means ± standard error (n = 3). The asterisk means a significant difference (* *p* < 0.05, *** *p* < 0.001) between sample groups at the same storage day.

The synthesis of astragalosides begins with cycloastragenol. UGT15 catalyzes the glycosylation of cycloastragenol to form cycloastragenol-3-O-β-D-xyloside. Cycloastragenol-3-O-β-D-xyloside not only forms astragaloside IV but also forms astragaloside III under the action of glycosyltransferase. Astragaloside IV is further oxidized to astragaloside II and astragaloside I. In this study, the ozone treatment group had higher contents of astragaloside I than the control group. We speculated that the ozone oxidizes the hydroxy group (-OH) in the pyranoid ring of astragaloside IV to form astragaloside II, which is further converted into astragaloside I (Figure 8); this is the main reason for why the content of astragaloside IV was significantly lower than in the control group.

## 5. Conclusions

**Figure 9:**
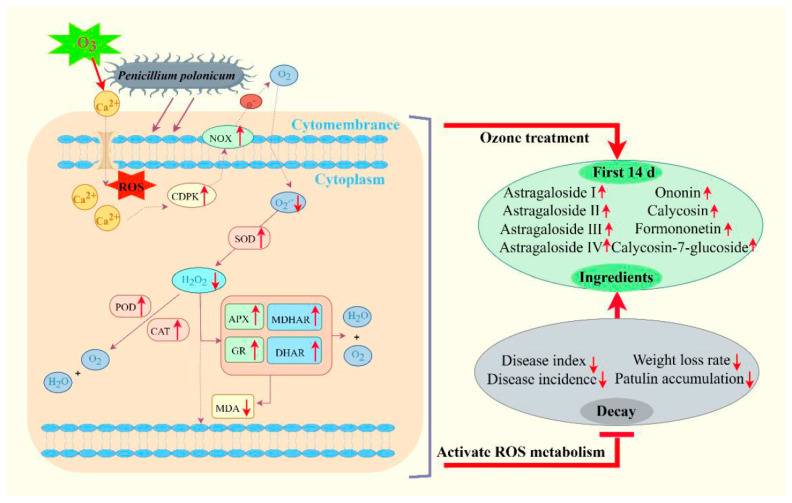
The possible mode of action of ozone treatment (compared with control group).

The results of this study show that ozone treatment effectively reduced the disease incidence, disease index, and PAT accumulation, decelerated the weight loss rate, and maintained the main ingredient contents in fresh *R. astragali*, which was attributed to activating ROS metabolism, enhancing the activities of NOX, SOD, CAT, POD, APX, MDHAR, DHAR, and GR, slowing down the production rate of O_2_^−^· and the content of H_2_O_2_ in *R. astragali*, inhibiting the accumulation of MDA, maintaining the integrity of the cell membrane, and preventing the infection of *P. polonicum* in *R. astragali*, Importantly, ozone treatment better maintained the active component content in the fresh *R. astragali* during storage; in particular, ozone treatment significantly delayed the decrease in the contents of astragaloside I, calycosin-7-glucopyranoside, and ononin by some chemical structure modification (Figure 9 presents the possible mode of action of ozone treatment). This study provides a theoretical basis for controlling postharvest diseases of Chinese medicinal materials. However, how to change the main active ingredients under the action of ozone needs further analysis.

## Data Availability

The original contributions presented in this study are included in the article. Further inquiries can be directed to the corresponding authors.

## Supplementary Materials

The following supporting information can be downloaded at: https://www.mdpi.com/article/10.3390/jof11060402/s1, Table S1: Contents of 10 active ingredients in *Radix astragali*.
