# Supplementary material for: Ozone Treatment Inhibited the Blue Mold Development and Maintained the Main Active Ingredient Content in Radix astragali Infected by Penicillium polonicum Through Activating Reactive Oxygen Species Metabolism
Source: J Fungi (Basel). 2025 May 23;11(6):402. doi: 10.3390/jof11060402 (PMC12194469; doi:10.3390/jof11060402)
Supplement: Supplementary file 1 [file jof-11-00402-s001.zip › jof-3580100-supplementary.pdf]

Table S1 Contents of 10 active ingredients in *Radix Astragali*

Ingredients / Time (d)		content（ng/ml）									
		1	2	3	4	5	6	7	8	9	10
CK	7	6343.3±229.9	651.1±37.9	495.7±7.5	485.8±7.6	95.9±3.5	481.1±17.9	131.1±4.1	28.6±0.9	1709.1±72.3	19.4±0.6
	14	4345.1±71.1	816.5±41.6	383.8±5.7	373.8±5.8	171.1±7.9	536.5±13.1	79.5±2.8	42.2±1.7	2920.8±134.2	61.9±1.6
	28	2684.1±207.7	487.3±12.7	358.1±8.4	348.1±8.3	178.8±1.7	727.1±21.2	332.4±8.5	87.2±3.2	2493.2±79.1	168.5±5.2
	42	1304.1±67.1	2232.5±61.8	3025.5±24.9	3019.1±24.9	1073.4±34.1	249.9±6.5	63.7±1.8	331.9±13.2	3708.2±147.4	589.1±19.7
	56	116.1±2.1	688.6±32.7	3015.4±56.4	3008.9±56.5	525.8±4.5	110.3±5.7	44.5±1.1	166.3±6.5	2875.4±130.4	298.9±9.5
O_3-1h	7	10603.8±209.4	923.5±56.7	589.6±9.6	579.8±9.7	185.6±3.8	561.4±9.7	273.3±6.5	97.9±3.7	2130.3±94.5	55.2±1.4
	14	5985.3±80.3	762.3±48.1	358.4±22.5	348.4±22.6	106.9±6.4	358.1±6.9	22.6±0.7	32.6±1.2	1123.3±85.3	30.1±1.1
	28	5152.8±90.8	540.6±30.3	522.8±6.5	513.1±6.7	73.67±1.5	442.1±12.2	75.2±2.2	14.8±0.5	1443.2±77.3	21.9±0.8
	42	4384.1±62.1	911.1±21.8	729.9±25.5	720.4±25.6	196.3±3.7	406.70±7.1	69.1±2.1	48.4±1.9	2784.5±93.3	124.2±5.8
	56	288.9±21.1	1055.4±80.8	2186.1±32.1	2178.5±32.1	412.8±3.7	193.6±2.1	23.2±0.8	135.7±5.5	2694.7±116.9	372.4±11.4
O_3-2h	7	11452.4±310.3	910.7±25.4	525.8±15.2	516.1±15.8	110.2±4.7	1010.2±26.4	222.2±5.8	12.7±0.4	4537.1±208.2	94.8±1.5
	14	9333.5±93.3	893.8±60.2	611.7±17.1	602.1±17.2	383.8±12.3	627.8±9.6	198.6±5.3	117.9±3.8	2633.2±106.2	182.1±4.1
	28	6605.7±151.5	787.3±67.8	541.5±8.4	531.8±8.5	109.5±6.3	416.1±8.9	100.8±3.6	33.6±1.3	2247.8±95.4	115.1±3.8
	42	4485.7±86.7	1410.1±13.1	1512.6±37.2	1504.1±37.4	171.1±9.6	304.7±3.6	64.8±1.9	43.5±1.8	2086.8±96.2	106.3±3.2
	56	2619.7±19.7	368.1±6.9	342.4±9.3	332.4±9.1	65.21±1.6	414.3±6.3	212.4±5.6	17.5±0.7	873.6±39.8	1.6±0.03

Note：1: AstragalosideI, 2: AstragalosideII, 3: Astragaloside III, 4: Astragaloside IV, 5: Calycosin, 6: Calycosin-7-glucoside, 7: Ononin, 8: Formononetin, 9: 7,2'-Dihydroxy-3',4'-Dimethoxyisoflavan, 10: 3-Hydroxy-9,10-Dimethoxypterocarpan.
